# Supplementary material for: MetaRibo-Seq measures translation in microbiomes
Source: Nat Commun. 2020 Jun 29;11:3268. doi: 10.1038/s41467-020-17081-z (PMC7324362; doi:10.1038/s41467-020-17081-z)
Supplement: Supplementary file 10 — Supplementary Data 7 [file 41467_2020_17081_MOESM10_ESM.zip › File2/Confidence_High_Taxonomy/430553_out.krona.html]

Javascript must be enabled to view this page.

members
magnitude
magnitudeUnassigned
count
unassigned
taxon
rank

430553\_out

4

2
superkingdom
4

4
phylum
976

class
200643
4

order
171549
4

4
171552
family

genus
838
4

2292365
species

SRS015794\_contig\_number\_2906SRS023526\_contig\_number\_22608SRS077641\_contig\_number\_15605SRS077849\_contig\_number\_10539
4
